# Short-Read and Long-Read Whole Genome Sequencing for SARS-CoV-2 Variants Identification

**DOI:** 10.3390/v17040584

**Published:** 2025-04-18

**Authors:** Mengfei Peng, Morgan L. Davis, Meghan L. Bentz, Alex Burgin, Mark Burroughs, Jasmine Padilla, Sarah Nobles, Yvette Unoarumhi, Kevin Tang

**Affiliations:** 1 Division of Core Laboratory Services and Response, Office of Laboratory Systems and Response, Centers for Disease Control & Prevention, Atlanta, GA 30329, USA; vjb0@cdc.gov (M.P.);; 2 Association of Public Health Laboratories, Bethesda, MD 20814, USA

**Keywords:** SARS-CoV-2, genomic surveillance, whole-genome sequencing, variants, lineage, sequencing depth, genome coverage

## Abstract

Genomic surveillance of SARS-CoV-2 is crucial for detecting emerging variants and informing public health responses. Various sequencing technologies are used for whole genome sequencing of SARS-CoV-2. This cross-platform benchmark study applied established bioinformatics tools to assess and improve the performance of Illumina NovaSeq, Oxford Nanopore Technologies MinION, and Pacific Biosciences Sequel II sequencing platforms in identifying SARS-CoV-2 variants and lineage assignment. NovaSeq produced the highest number of reads and bases, depth of coverage, completeness of consensus genomes, stable mapping coverage across open reading frames in the genome, and consistent lineage assignments. The long-read sequencing platforms had lower yields, sequencing depth, and mapping coverage, limiting the number of qualified sequences for lineage assignment and variant identification. However, implementing proper quality controls on sequence data overcame these limitations and achieved consistent SARS-CoV-2 lineage assignments across all three sequencing platforms. The advancements in library preparation and technology for long-read sequencing are likely to enhance sequence quality and expand genome coverage, effectively addressing current limitations in genome analysis. By merging the unique advantages of both short- and long-read methods, we can significantly improve SARS-CoV-2 genomic surveillance and provide insights into sequencing strategies for other RNA viruses, pending further validation. This may lead to precise tracking of viral evolution and support public health policy decisions.

## 1. Introduction

The coronavirus disease 2019 (COVID-19), declared a global pandemic by the World Health Organization (WHO) in 2020, continues to circulate around the world with substantial incidence despite ongoing vaccination [1]. Although the causative agent, severe acute respiratory syndrome coronavirus 2 (SARS-CoV-2), has a relatively lower mutation rate in comparison with many other RNA viruses, it keeps evolving into variants of concern with higher transmissibility and abilities to evade vaccine-induced immunity [2,3]. Consequently, genomic surveillance of SARS-CoV-2 is critical over the course of the pandemic as new variants emerge. An efficient and accurate SARS-CoV-2 genomic surveillance can facilitate early detection of the variants of concern, tracing viral transmission at the population level and informing public health decisions of preventive strategies [4]. To urgently establish a global SARS-CoV-2 surveillance system, the World Health Organization has recommended that all countries conduct genomic sequencing of a minimum of 1% of their infections. In this context, more than 17 million SARS-CoV-2 genomic sequences have been deposited in the GISAID repository (https://gisaid.org; accessed in March 2025) for public availability.

A broad range of sequencing technologies, including whole genome sequencing (WGS), amplicon-based sequencing, and shotgun metagenomics, have been developed and are currently used to sequence SARS-CoV-2 strains [5,6,7]. The short-read sequencing technology by Illumina, long-read Oxford Nanopore Technologies (ONT) sequencing, and long-read Pacific Biosciences (PacBio) sequencing technology are among the most commonly and widely applied sequencing technologies by public health laboratories worldwide [8,9]. Each method has varying accuracy, sensitivity, and reproducibility of sequencing performance which has been previously studied and compared [10,11]. Considering the continuous emergence and spread of new SARS-CoV-2 sub-lineages, WGS is crucial for identifying emerging variants and detection of viral mutations of concern. Furthermore, variations in sequencing performance and variant identification among WGS platforms have been reported with highly controversial comments; some suggested that long-read sequencing can resolve variants better, while others argued that short-read sequencing is more reliable for detecting low-frequency variants [12,13,14]. However, only limited studies have evidently defined the quality control inclusive criteria for SARS-CoV-2 genomic data across the short-read and long-read sequencing platforms.

This cross-platform benchmark study investigates the commonly used three WGS platforms, Illumina NovaSeq, ONT MinION, and PacBio Sequel II, based on sequencing 92 SARS-CoV-2-positive clinical samples. Multiple library preparation, sequencing performance, and practical parameters are assessed across different sequencing platforms. These include, but are not limited to, read numbers, sequencing depth, genome coverage, variant calling, sequence ambiguity, and clade and lineage assignments. While several studies have evaluated SARS-CoV-2 sequencing performance across different platforms [12,13,15], our study uniquely establishes stringent quality control criteria across short-read and long-read sequencing technologies. By systematically benchmarking sequencing depth, mapping coverage, and lineage consistency across platforms, we provide actionable recommendations for improving SARS-CoV-2 genomic surveillance and variants detection efforts by public health laboratories.

## 2. Materials and Methods

### 2.1. Total Nucleic Acids (TNA) Extraction

TNA extractions were performed on 92 SARS-CoV-2 positive samples by the Core Operation and Outbreak Response Laboratory at the United States Centers for Disease Control and Prevention (CDC), Atlanta, Georgia, USA. Using a 96-well MP96 processing cartridge, 100 µL of each sample was added to 350 µL of MagNA Pure 96 (MP96) External Lysis Buffer and incubated at room temperature for 10 min for virus inactivation. After the incubation, the cartridge was loaded onto an MP96 extraction platform and TNA was extracted using the MagNA Pure 96 DNA and Viral NA Small Volume Kit (Roche, Indianapolis, IN, USA). All the TNA samples were used for further processing and sequencing on different platforms following standardized library preparation protocols to ensure comparability across platforms.

### 2.2. Genomic Sequencing by Illumina NovaSeq Platform

cDNA was synthesized using the SuperScript IV 1st strand synthesis system (Thermo Fisher Scientific, Waltham, MA, USA) with the following thermal cycler conditions: 25 °C for 10 min, 50 °C for 30 min, and 80 °C for 10 min. Libraries were constructed, normalized, and pooled using IDT’s xGen Amplicon Core Kit for SAR-CoV2 (Integrated DNA Technologies, Ann Arbor, MI, USA) following the manufacturer’s instructions. The final 2 nM pool was sequenced on Illumina NovaSeq (Illumina, San Diego, CA, USA) using NovaSeq 6000 SP Reagent Kit v1.5 (300 cycles).

### 2.3. Genomic Sequencing by ONT MinION Platform

TNA was reverse transcribed and then amplified by PCR with the Midnight RT PCR Expansion (EXP-MRT001; Oxford Nanopore Technologies, Oxford, UK). The reverse transcription reaction was performed at 25 °C for 2 min, 55 °C for 10 min, and 95 °C for 1 min. PCR amplification began with an initial denaturation at 95 °C for 30 s, followed by 35 cycles of denaturation at 98 °C for 15 s, annealing and extension at 61 °C for 2 min and 65 °C for 3 min. The primer pools were combined, and libraries were prepared using the rapid barcoding kit (SQK-RBK110.96) and sequenced on MinION [16].

### 2.4. Genomic Sequencing by PacBio Sequel II Platform

TNA was transformed into cDNA using the Molecular Loop Viral RNA Capture Kit (ML5200-PB; Molecular Loop, Woburn, MA, USA) with the following thermal cycler conditions: 25 °C for 10 min, 50 °C for 50 min, and 95 °C for 1 min. This was followed by 24 h probe hybridization at 55 °C and barcoded using M13 barcodes (102-135-500; PacBio, Menlo Park, CA, USA) for amplification with 26 cycles of 95 °C for 3 min, 98 °C for 15 s, 55 °C for 15 s, and 72 °C for 90 s. The cDNA samples were then pooled in equal volumes and purified using Promega ProNex beads (PRNG2001; Fisher Scientific, Waltham, MA, USA). Libraries were prepared from the barcoded cDNA using SMRTbell Express Template prep kit 2.0 (100-938-900; PacBio, Menlo Park, CA, USA). The libraries were prepared for sequencing using the Sequel binding kit 2.1 (101-820-500; PacBio, Menlo Park, CA, USA) and sequenced on the Sequel II instrument.

### 2.5. Quality Control and Trimming

The raw sequencing data generated by different sequencing platforms were in the form of FASTQ files for follow-up processing and analyses. Trimming per read of the collected raw reads was performed with fastp (v0.23.4; Chinese Academy of Sciences, Shenzhen, China) [qualified_quality_phred:15; unqualified_percent_limit:40; n_base_limit:5; complexity_threshold:30; poly_g_min_len:10; poly_x_min_len:10; cut_mean_quality:20; overrepresentation_sampling:20] in adapter sequences auto-detection mode (detect_adapter). All reads were filtered with a minimum length of 40 bp. The quality of FASTQ files was examined by FastQC (v0.11.5; Babraham Institute, Cambridge, UK), and the FastQC reports were aggregated with MultiQC (v1.24; SciLifeLab, Stockholm, Sweden) for various analyses.

### 2.6. Genome Mapping and Variant Calling

The trimmed and filtered FASTQ files were collected and mapped against a SARS-CoV-2 reference genome (Wuhan-Hu-1/2020; NC_045512) by minimap2 (v2.17; Broad Institute of MIT and Harvard, Cambridge, MA, USA) -x sr for Illumina NovaSeq, map-ont for ONT MinION, and map-pb for PacBio Sequel II reads. Using Samtools (v1.18; Broad Institute of MIT and Harvard, Cambridge, MA, USA), SAM files from mapping were converted to BAM files and sorted. Variants in the sorted BAM files were further called and identified by bcftools (v1.10.2; Broad Institute of MIT and Harvard, Cambridge, MA, USA). SARS-CoV-2 consensus genomes were generated with the Iterative Refinement Meta-Assembler (IRMA, v1.1.3; Centers for Disease Control and Prevention, Atlanta, GA, USA) approach [17] and pipeline (MODULE: CoV; MATCH_PROG: BLAT; ALIGN_PROG: SAM; SORT_PROG: BLAT; ASSEM_PROG: MINIMAP2; MAX_ROUNDS: 5). The passing metrics for the sequencing data were defined as: (1) percent ambiguous nucleotides (N) < 10%; (2) reference genome coverage > 90%.

### 2.7. Phylogenetic Placement and Clade/Lineage Assignment

The consensus sequences from different sequencing platforms were collected and aligned with the SARS-CoV-2 reference NC_045512 using MAFFT (v7.520; Osaka University, Osaka, Japan), followed by phylogenetic placement by using FastTree (v2.1.11; Lawrence Berkeley National Laboratory, Berkeley, CA, USA). The aligned sequences were further analyzed with Nextclade (v3.7.1; Nextstrain, Seattle, WA, USA) for Nextstrain clade determination and lineage assignment, as well as through implementing the dynamic nomenclature of SARS-CoV-2 lineages (Pango nomenclature) using Pangolin (v4.3.1; University of Edinburgh, Edinburgh, UK) [max-ambig:0.3; min-length:25,000] in the accurate and stable analysis mode (pUShER inference). The assigned lineages were collected for all the samples for comparison among different sequencing platforms.

### 2.8. Statistical Analyses and Visualization

Kruskal–Wallis tests were initially employed to determine the potential significant differences in the mapping coverages at the SARS-CoV-2 genome and individual open reading frame (ORF) gene regions as well as the numbers of mutations, frame shifts, and ambiguous nucleotides in the SARS-CoV-2 consensus genome sequences among Illumina NovaSeq, ONT MinION, and PacBio Sequel II sequencing platforms. These were followed by pair-wise comparisons between sequencing platforms employing Mann–Whitney tests. Significance was determined based on a *p*-value less than 0.05. All the statistical analyses and figures generation were carried out using R (v4.3.2; R Foundation, Indianapolis, IN, USA).

## 3. Results

### 3.1. Sequencing Quality Statistics

As shown in Table 1, a total of 16,889.40 Mbp and 261,428,474 reads were yielded from sequencing on the Illumina NovaSeq platform, with an average of 2,841,614 reads per sample. NovaSeq achieved an average base (Phred) quality score (probability of error per base call in a log scale) of 35.9, which is between 1 in 1000 to 1 in 10,000 probabilities of calling an incorrect base (99.9–99.99% accuracy). The average NovaSeq sequence length was 117 bp. A total of 576.16 Mbp and 932,604 reads were yielded from sequencing on the ONT MinION platform, with an average of 10,137 reads per sample. The average MinION sequence length was 584 bp, and 97.83% of the samples achieved an average base quality score higher than 20 (Q20). A total of 505.91 Mbp and 630,330 reads were yielded from sequencing on the PacBio Sequel II platform, with an average of 6851 reads per sample. The average Sequel II sequence length was 1129 bp per sample, and all the samples achieved an average base quality score higher than 30 (Q30). Overall, NovaSeq generated higher yield and total reads, while Sequel II sequences had longer reads; all sequences from these two platforms achieved a 100% Q30 score.

### 3.2. Sequence Pass Rate and Lineage Assignment Inconsistency

**Figure 1:**
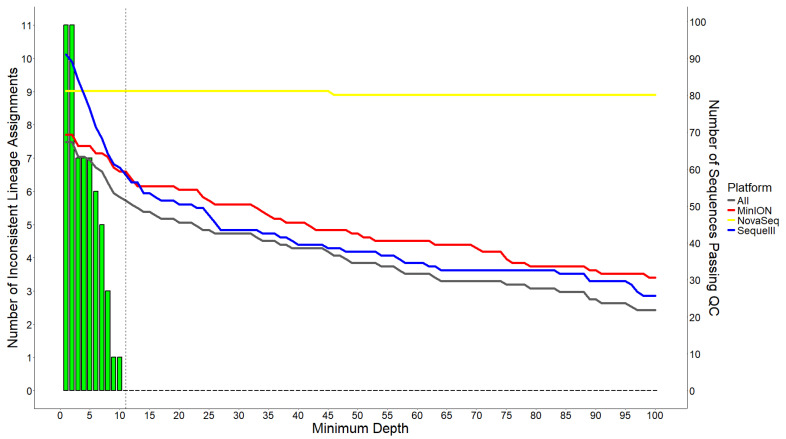
Lineage assignment inconsistency and qualified SARS-CoV-2 sequences across sequencing platforms. The bars show the number of samples with inconsistent lineage assignment across the three sequencing platforms. The lines show the number of qualified sequences from individual sequencing platforms and from all three sequencing platforms, with passing metrics of <10% ambiguous nucleotides and >90% genome coverage based on the minimum coverage depth. The vertical dashed line indicates the minimum coverage depth (11×) ensuring consistent lineage assignments for qualified sequences.

We set the initial quality control criteria to require sequences with <10% ambiguous nucleotides and >90% genome coverage. The pass rates for sequences are 89.13% for NovaSeq, 76.09% for MinION, and 100.00% for Sequel II (Table 1). However, minimal sequencing depth can affect the accuracy of the sequence data, leading to an increased risk of failing quality criteria. The impacts of minimal sequencing depths on the passing rate of SARS-CoV-2 genome sequences and their SARS-CoV-2 lineage assignment are illustrated in Figure 1. After the minimal depth is applied, the number of passing NovaSeq sequences slightly decreased from 82 at 1× sequencing depth to 81 within the minimum depth range of 46× to 100×. The passing rate of Sequel II sequences decreased significantly from 100% (92/92) to 66.30% (61/92) at 10× depth, and further dropped to 28.26 (26/92) at 100× depth; similarly, the MinION sequences had a passing rate of 76.09% (70/92) at a minimum depth of 1×, and the rate decreased with higher depth, dropping to 33.70% (31/92) at the minimum of 100× depth. Overall, the number of samples with sequences from all three sequencing platforms passing the metrics was primarily limited by the qualified long-read sequences, which decreased from 68 at a minimum of 1× sequencing depth to 22 at a minimum of 100× sequencing depth.

We examined the consistency of SARS-CoV-2 lineage assignment across the NovaSeq, MinION, and Sequel II sequencing platforms. Inconsistently assigned lineages were observed with low (1× to 10×) minimum sequencing depths that defined the genome coverage passing metric (Figure 1). A total of 11 samples were found with inconsistent SARS-CoV-2 lineages assigned across the three sequencing platforms at 1× depth. This inconsistent number decreased with the higher minimum depth and reached 0 at > 10× depth, where consistent lineage assignments were obtained for the 52 qualified samples across all platforms. These results suggest that low sequencing depth (≤10×)-based genome coverage passing metrics may lead to inaccurate SARS-CoV-2 lineage assignment and variant identification.

### 3.3. Mapping Quality and Genome Coverage

**Figure 2:**
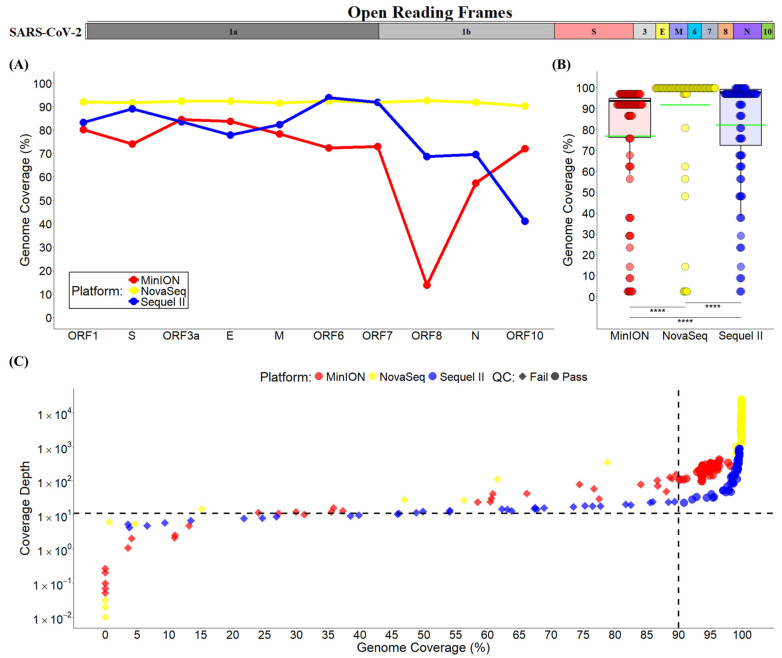
Percent mapping coverages across sequencing platforms. (**A**) The average mapping coverages based on > 10× sequencing depth across all 10 open reading frames (ORFs) of the SARS-CoV-2 genome within each sequencing platform. (**B**) Comparison of all-sample genome coverages among the three sequencing platforms. The green lines represent the mean mapping coverages for each individual sequencing platform. ****, *p* < 0.0001 between groups as indicated. (**C**) Distributions of coverage depth and genome coverage for all samples across the three sequencing platforms. The dashed lines represent >10× sequencing depth and >90% genome coverage.

The mapping quality with the SARS-CoV-2 reference NC_045512 was examined based on >10× sequencing depth. Reads from the NovaSeq platform had a mean sequencing depth of 9643 and an average percent mapping coverage of 91.83% (Table 1). Meanwhile, reads from long-read sequencing platforms had lower sequencing depth (MinION: 157; Sequel II: 177) and mapping coverage (MinION: 76.91%; Sequel II: 82.22%). The percent mapping coverages of the SARS-CoV-2 genome and each individual ORF gene region were evaluated across the three sequencing platforms (Figure 2). The Illumina NovaSeq reads obtained stable mean mapping coverages across all 10 ORFs (Figure 2A), with the highest mean genome coverage (91.83% ± 27.41%; *p* < 0.0001; Figure 2B) among all the sequencing platforms; the long reads from ONT MinION had the lowest mean genome mapping coverage (76.91% ± 28.55%%; *p* < 0.0001; Figure 2B).

The NovaSeq reads had the highest mean mapping coverages (~90%, Figure 2A) across all ORFs (*p* < 0.001), except for ORF6 where a relatively higher mean coverage was found with Sequel II reads (Supplementary Figure S1). The Sequel II and MinION reads had lower mapping coverage (70–90%) for the first 7 ORFs and even lower mapping coverages for the rest ORFs (Figure 2A; Supplementary Figure S1J). The lowest mean mapping coverage was obtained at the ORF8 regions by the MinION reads (Figure 2A; Supplementary Figure S1H). As shown in the detailed distributions of the percent genome coverage vs. sequencing depth (Figure 2C), most of the reads reached > 10× sequencing depth; however, the passing rate of the long-read sequences was compromised by their genome mapping coverage. Similar patterns were observed across all the 10 ORFs (Supplementary Figure S2).

### 3.4. Consensus Genome Ambiguity and Variant Calling

**Figure 3:**
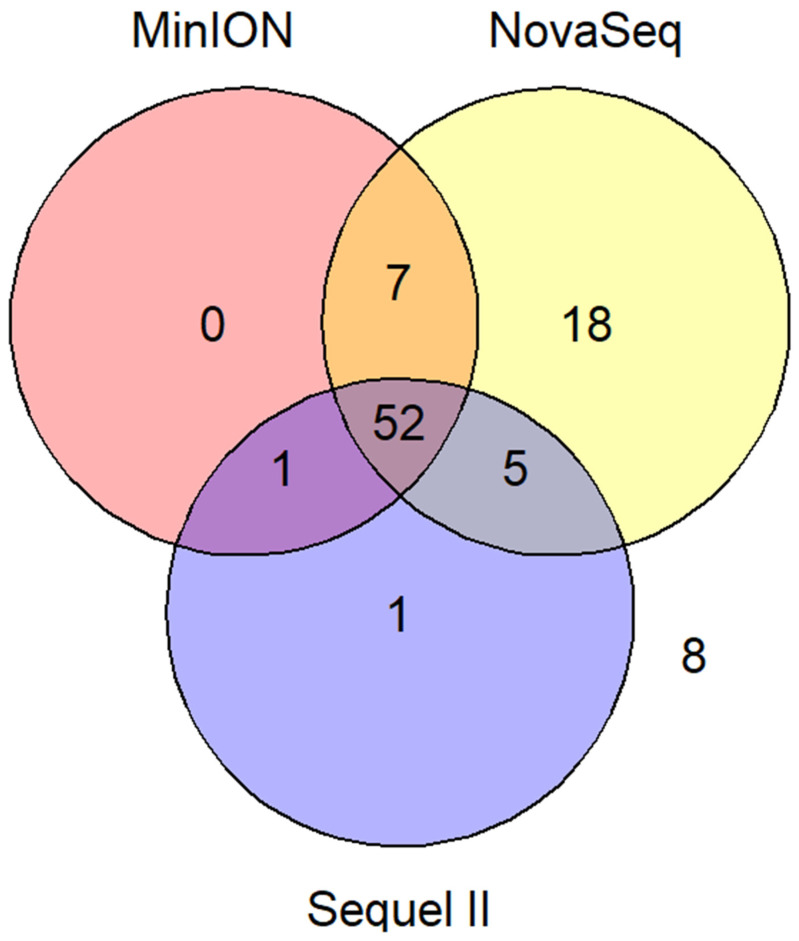
Relations and commonness of samples with qualified SARS-CoV-2 consensus genome sequences across sequencing platforms. The number (8) outside the Venn diagram elements represents the 8 samples with no qualified SARS-CoV-2 consensus sequences regardless of their sequencing platforms. To be noted, identical SARS-CoV-2 lineage assignment was obtained from 52 samples with qualified consensus genome sequences from all three sequencing platforms.

As illustrated in Figure 3, out of 92 total samples, the percentage of sequences qualified for SARS-CoV-2 lineage assignment was 89.13% (82 samples) for NovaSeq, 65.22% (60 samples) for MinION, and 64.13% (59 samples) for Sequel II (Table 1). Across all platforms, 52 samples (56.52%) had qualified sequences, while 8 samples (8.70%) had no qualified sequences on any platform. Of the remaining 32 samples, 13 were qualified on two platforms, 18 only on NovaSeq, and 1 only on Sequel II.

**Figure 4:**
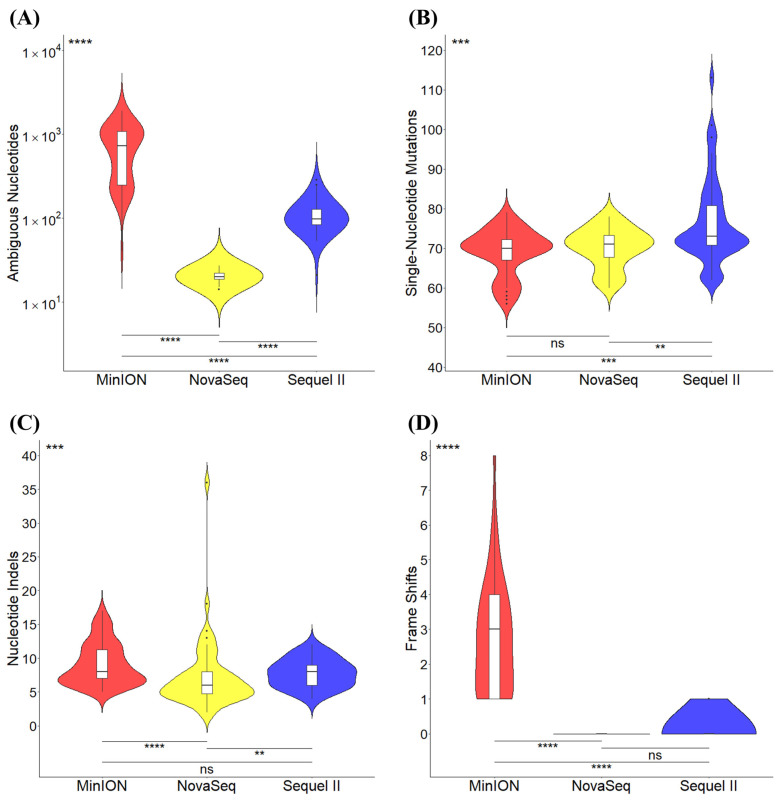
Comparisons of variant calling and nucleotide ambiguity across sequencing platforms. Violin plots displaying the number and density of (**A**) ambiguous nucleotides, (**B**) single-nucleotide mutations, (**C**) nucleotide indels, and (**D**) frameshift mutations called in the qualified SARS-CoV-2 consensus genome sequences of the 52 samples across all the sequencing platforms. Note: ns, non-significant; **, *p* < 0.01; ***, *p* < 0.001; ****, *p* < 0.0001 between groups as indicated.

The sequence ambiguity and variant mutations called from the qualified SARS-CoV-2 consensus genomes across all three sequencing platforms were further analyzed (Figure 4). Specifically, the largest (*p* < 0.0001) number of ambiguous nucleotides was found in the MinION sequences, while the NovaSeq sequences had the lowest (*p* < 0.0001) level of ambiguity (Figure 4A). Furthermore, a relatively larger averaged number of single-nucleotide mutations (SNPs) was called from the consensus sequences generated by the Sequel II platform, while similar median numbers of SNPs were observed among all three sequencing platforms (Figure 4B). Fewer (*p* < 0.01) nucleotide indel mutations were called from the NovaSeq sequences, while no significant (*p* > 0.05) difference was observed between the MinION and Sequel II platforms (Figure 4C). In addition, a significantly larger (*p* < 0.0001) number of frame shifts was observed in the MinION sequences, while no significant (*p* > 0.05) difference was observed between the NovaSeq and Sequel II platforms (Figure 4D). Overall, MinION sequences had the highest numbers of ambiguous nucleotides, indels, and frame shifts, posing potential issues on quality and accuracy.

### 3.5. Phylogenetic Placement of the SARS-CoV-2 Sequences

**Figure 5:**
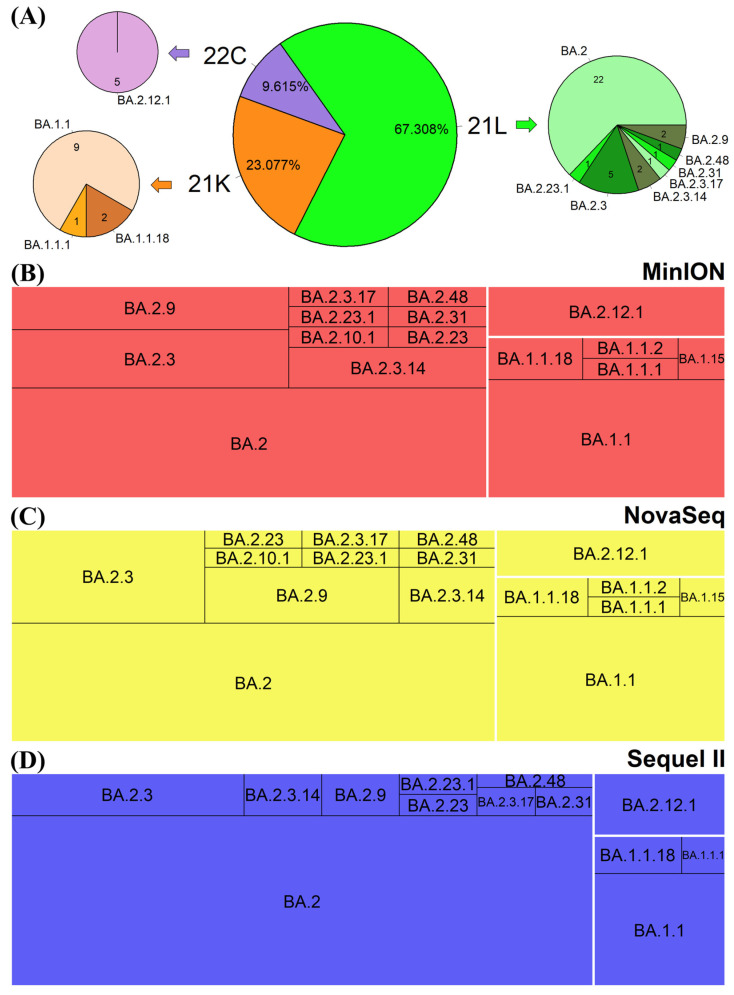
Phylogenetic distributions of the assigned SARS-CoV-2 genomes. (**A**) The main pie chart showing assigned Nextstrain SARS-CoV-2 clades for the 52 samples with qualified sequences across all three sequencing platforms, with extended pie charts showing Pango SARS-CoV-2 lineages assignments. (**B**–**D**) Tree maps showing the detailed Nextstrain SARS-CoV-2 clade and Pango SARS-CoV-2 lineages assignments for the 60, 82, and 59 qualified sequences from the Oxford Nanopore Technologies MinION, Illumina NovaSeq, and PacBio Sequel II sequencing platforms, respectively.

The qualified SARS-CoV-2 sequences were subjected to clade and lineage identification and a 100% rate of assignment was achieved for Nextstrain SARS-CoV-2 clades and lineages as well as Pango SARS-CoV-2 nomenclature (lineage). When comparing Nextclade and Pangolin lineage assignments, identical SARS-CoV-2 lineage assignment was obtained from the 52 samples with qualified consensus genome sequences shared among all three sequencing platforms. The phylogenetic distributions of the assigned SARS-CoV-2 genomes are illustrated in Figure 5. Among the 52 samples, clade 21L accounted for 67.31% (35/52) of the total, with 22 samples assigned to BA.2 lineage, followed by 5 samples to BA.2.3 lineage; clade 21K accounted for 23.08% (12/52) of the total, with 9 samples assigned to BA.1.1 lineage; clade 22C accounted for 9.62% (5/52) of the total, and all were assigned into BA.2.12.1 lineage (Figure 5A). To be noted, a total of 84 sequences from NovaSeq, 75 from MinION, and 92 from Sequel II could be assigned with a Nextclade or Pangolin lineage, among which BA.2 lineage was assigned with extremely higher predominance (65.22%) to the Sequel II sequences than to the sequences from the other platforms (NovaSeq: 38.10%; MinION: 34.67%) (Figure 5B–D). These indicate that low-quality Sequel II sequences tend to be phylogenetically misplaced into BA.2 lineage. Ensuring the sequence quality especially genome coverage at a minimum depth is necessary for accurate and consistent phylogenetic placement.

## 4. Discussion

As the COVID-19 pandemic continues worldwide due to the variants of concern and emerging novel variants [18], close genomic surveillance and monitoring of SARS-CoV-2 evolution remains crucial for sustained management of the pandemic and assessment of potential changes in pathogenicity and transmissibility [4,19]. Although various molecular approaches have been developed and employed for sequencing SARS-CoV-2, high-throughput WGS is widely recognized as the most accurate and efficient for variant detection [20,21]. The SARS-CoV-2 Sequencing for Public Health Emergency Response, Epidemiology, and Surveillance (SPHERES) initiative was established by the United States Centers for Disease Control and Prevention at the early stage of the COVID-19 pandemic to facilitate and accelerate real-time pathogen WGS. This cross-platform benchmarking study assessed the capability and accuracy of Illumina-based short-read sequencing and ONT/PacBio-based long-read sequencing for the detection of SARS-CoV-2 SNPs and viral lineages. Unlike prior studies that primarily focused on individual sequencing platforms [14,20,22], our study systematically evaluates the impact of sequencing depth and quality control criteria on SARS-CoV-2 lineage assignment across multiple platforms. These findings offer practical guidelines for improving SARS-CoV-2 surveillance by optimizing platform selection and bioinformatics analyses.

The Illumina platform is capable of simultaneously sequencing a large number of samples through multiplexing and generating high yields [23]. In comparison with long-read sequencing platforms examined in this study, the Illumina NovaSeq platform achieved high total reads, total bases, and depth of coverage for all sequenced samples. In comparison, long-read sequencing techniques can read longer lengths of sequences, which addresses the major challenges faced by short-read sequencing such as the detection of large structural variants or long repetitive sequences [24,25]. However, in comparison with short-read sequencing, long-read sequencing is associated with compromised sequencing depth and sequencing accuracy per read [26]. In this study, the long-read sequences were associated with higher numbers of ambiguous reads, lower sequencing depth, alongside lower mapping coverage. The numbers of qualified MinION and Sequel II sequences were mainly limited by their relatively low genome coverage defined at a minimum sequencing depth. Furthermore, we observed that the long-read sequences exhibited inconsistent mapping coverage throughout the ORFs, especially with lower mapping coverages in gene regions near the 3’ end. This was particularly demonstrated by the substantially low coverage of PacBio Sequel II sequences at the ORF10 region and the lowest coverage of ONT MinION sequences at the ORF8, compared with the other mapped regions of the SARS-CoV-2 genome. Although specific reasons for such inconsistency of mapping coverage remain unclear, these potentially pose challenges to long-read sequencing for effective and accurate SARS-CoV-2 lineage identification.

Among the multiple distinct lineages of Omicron SARS-CoV-2, lineage BA.2 has largely supplanted BA.1, the lineage of initial Omicron surge in 2021, worldwide since early 2022 [27]. The present study could successfully identify the SARS-CoV-2 lineages of all the WGS sequences, which were assigned in sublineages of either BA.1 or BA.2. Meanwhile, with ≤ 10× minimum depths defining the genome coverage passing metric, all the samples with conflicted lineage assignments across sequencing platforms were assigned into the BA.2 lineage with PacBio Sequel II sequencing platform, while consistent assignments into the sublineages or parent lineages were observed between NovaSeq and MinION sequencing platforms (Supplementary Tables S1–S3). The Pango nomenclature classifies complete or near-complete SARS-CoV-2 genomes, while a less complete genome can be assigned by Pangolin through inference and estimation of a known lineage to which the query genomic sequence most likely belongs [28]. Furthermore, pUShER identifies the lineages by performing phylogenetic placements with a maximum parsimony approach [29]. Therefore, inconsistency of Pango lineage assignments is more closely associated with phylogenomic inference rather than varying genome ambiguity, while confidence in identifying single-nucleotide variants highly replies on the correspondent sequencing depth. When comparing the 11 sequences with inconsistent lineage assignments across sequencing platforms, the Sequel II sequences had the lowest coverage depth at most of the SNP sites (Supplementary Table S4), inferring the least confidence in calling these single-nucleotide variants; more consistent SNPs were called from the NovaSeq and MinION sequences, especially in the crucial ORF1a polyprotein gene (266-13483 bp) and surface glycoprotein gene (21563-25384 bp) regions (Supplementary Figure S3) at which diverse prevalent mutation patterns have been identified among Omicron BA.1 and BA.2 lineages [30,31]. As a result, the MinION and NovaSeq sequences were found more phylogenetically related (Supplementary Figure S3), presenting identical lineage assignments between the platforms.

High-depth WGS serves as the golden standard of DNA sequencing due to its ability to interrogate the highest proportion of genetic variations in the genome. Low coverage depth usually introduces sequence errors which compromise the accuracy of downstream analyses [32]. The range of mapping coverage depth has been widely studied and recommended for accurate variant detection and assembly of the human genome, for which an average depth of 35× to 50× for next-generation sequencing and >15× for HiFi third-generation sequencing are established for reliable calling of single-nucleotide variants and small indels across 95% of the genome [33,34]. Comprehensive studies or recommendations are not available yet for viral genome assembly and lineage detection. The current study determined relatively strict passing metrics with genome coverage at a minimum of >10× sequencing depth for both short- and long-read sequences, slightly sacrificing the number of qualified samples, successfully minimizing potential lineage assignment inconsistency, and achieving equivalent quality, especially for long-read sequencing. Future studies on the sensitivity–specificity and cost-efficiency of short-read and long-read sequencing under different genome coverage and sequencing depth thresholds could further characterize and optimize the quality control criteria for SARS-CoV-2 genomic surveillance.

When utilizing publicly available SARS-CoV-2 genomic data (e.g., from GISAID), researchers should be aware of potential biases related to the sequencing platform [35]. We recommend applying standardized quality control filters to ensure the comparability of sequences across platforms. Additionally, implementing platform-aware bioinformatics analyses, such as hybrid approaches that integrate both short-read and long-read data, can further improve the accuracy of evolutionary studies [35,36]. One limitation of our study is the observed discrepancy in SNP detection across sequencing platforms, particularly the relatively higher average number of SNPs identified in PacBio Sequel II sequences. This could be due to differences in the inherent error profile of PacBio HiFi reads [37], potential base-calling errors (especially in homopolymer regions) [38], and variations in sequence coverage across ORFs. Future studies should further investigate these discrepancies by validating SNP calls using orthogonal methods such as Sanger sequencing or hybrid assemblies that combine short- and long-read data [39,40].

## 5. Conclusions

This study underscores the critical role of high-throughput WGS in SARS-CoV-2 genomic surveillance, assessing Illumina NovaSeq, ONT MinION, and PacBio Sequel II sequencing platforms for their efficacy in SARS-CoV-2 variant identification. Illumina NovaSeq sequences achieved high yields and sequencing depth, stably high mapping coverages across all ORFs, and consistent Pangolin lineage assignment, making it ideal for large-scale COVID-19 surveillance efforts. The long-read sequencing platforms, especially ONT MinION, present challenges particularly notable for small genomes, including fewer read numbers, lower depths of sequencing, and inconsistent mapping coverage over ORFs. Lineage assignment analysis revealed no discrepancy across sequencing platforms at passing metrics based on > 10× sequencing depths, while such consistency necessitates a minimum depth requirement for long-read sequences. We emphasize continued investment in genomic surveillance infrastructure and technology development that is essential for staying ahead of emerging variants and informing public health responses. It should be noted that since the collection of these data, PacBio has upgraded the chemistry of sequencing and library preparation kits to a new version (v3). Our ability to monitor SARS-CoV-2 evolution and mitigate the impact of future outbreaks can be further enhanced by leveraging the strengths of short-read and long-read sequencing platforms and optimizing bioinformatic analysis pipelines. Further research is needed to determine the applicability of these findings to other viral pathogens.

## Figures and Tables

**Table 1:** Summary of sequencing quality, genome mapping, and variant calling statistics.

Sequencing Platform	TOTAL					AVERAGE						
	Yield (Mbp)	Reads	%Q20	%Q30	%Passing QC Rate ^1^	Read Length (bp)	%Genome Coverage	Depth	%N	SNPs	Indels	FSs
NovaSeq	16,889.40	261,428,474	100.00	100.00	89.13% (82/92)	117.42	91.83	9642.84	6.98	66.82	6.75	0.10
MinION	576.16	932,604	97.83	0.00	76.09% (70/92)	584.02	76.91	157.10	15.70	58.79	9.01	4.68
Sequel II	505.91	630,330	100.00	100.00	100.00% (92/92)	1129.45	82.22	176.85	0.95	90.55	8.26	0.60

^1^ Passing Rate based on initial quality control criteria: <10% ambiguous nucleotides and >90% genome coverage at sequencing depth > 0×. Note: %N, percent ambiguous nucleotides; SNPs, single-nucleotide mutations; FSs, frameshift mutations.

## Data Availability

The original contributions presented in the study are included in the article/Supplementary Materials, further inquiries can be directed to the corresponding author.

## Supplementary Materials

The following supporting information can be downloaded at: https://www.mdpi.com/article/10.3390/v17040584/s1, Figure S1: Mapping coverages across all 10 open reading frames (ORFs) of the SARS-CoV-2 genome; Figure S2: Distributions of coverage depth and genome coverage based on >10× sequencing depth across all 10 open reading frames (ORFs) of the SARS-CoV-2 genome; Figure S3: Phylogeny and single-nucleotide variants of SARS-CoV-2 consensus genomes with inconsistent lineage assignments across sequencing platforms; Table S1: Illumina NovaSeq sequencing quality, genome mapping, and variant calling statistics; Table S2: Oxford Nanopore Technologies MinION sequencing quality, genome mapping, and variant calling statistics; Table S3: Pacific Biosciences Sequel II sequencing quality, genome mapping, and variant calling statistics; Table S4: Sequencing depths at genomic positions with single-nucleotide mutation calling.
