# Supplementary figures and images for: Short-Read and Long-Read Whole Genome Sequencing for SARS-CoV-2 Variants Identification
Source: Viruses. 2025 Apr 18;17(4):584. doi: 10.3390/v17040584 (PMC12031342; doi:10.3390/v17040584)

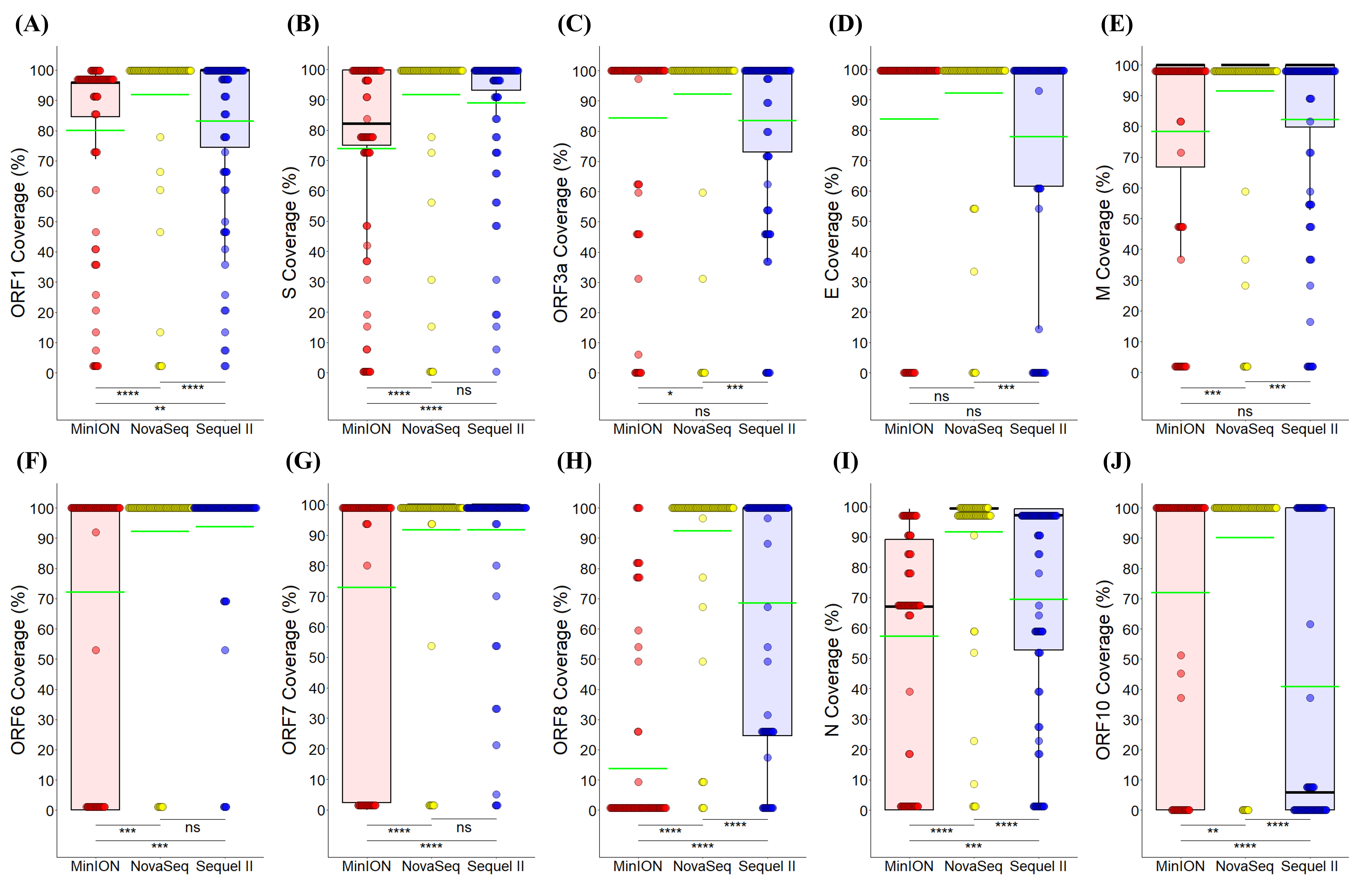

Supplement: Supplementary file 1 [file viruses-17-00584-s001.zip › Figure S1.png]

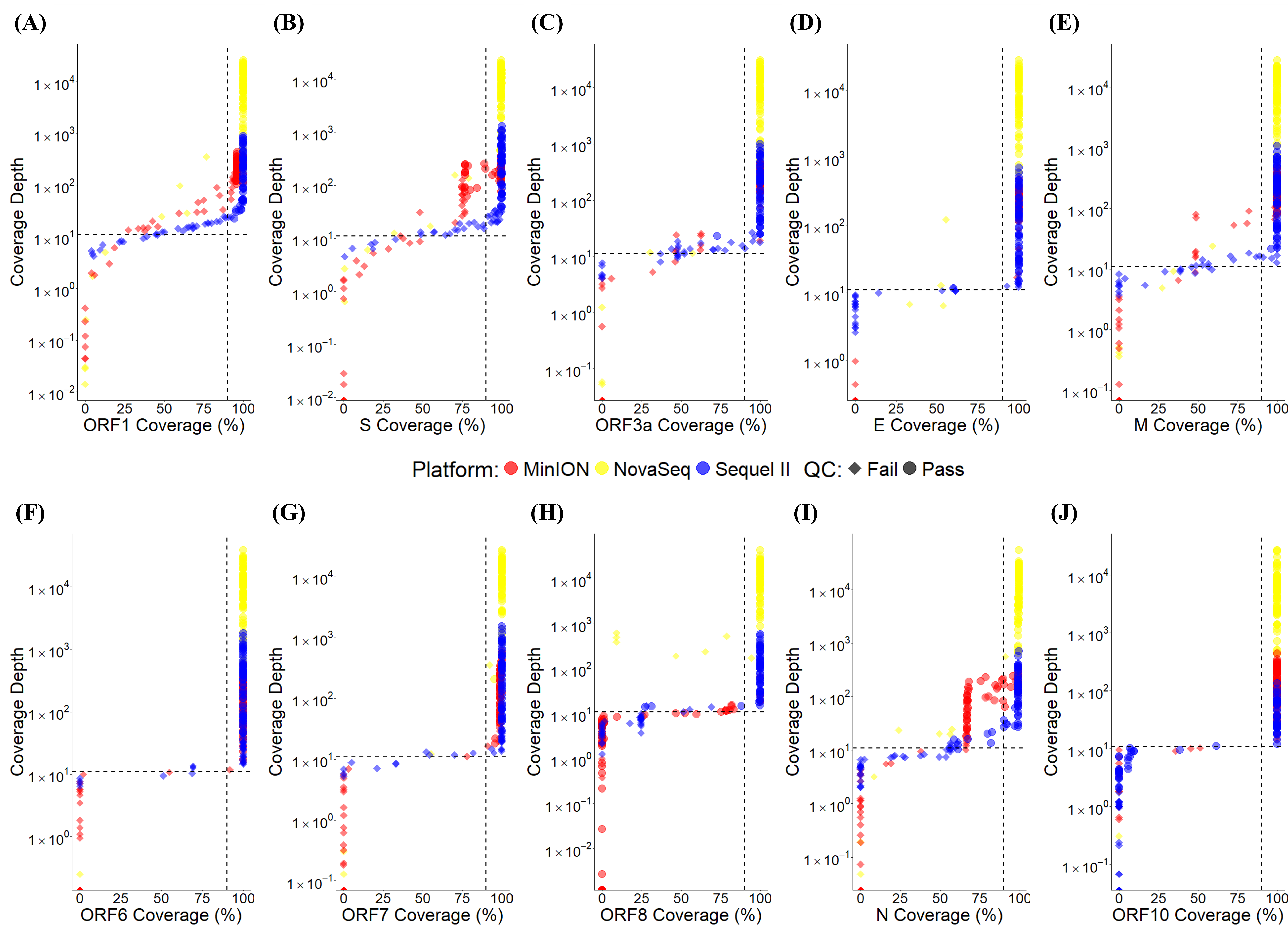

Supplement: Supplementary file 1 [file viruses-17-00584-s001.zip › Figure S2.png]

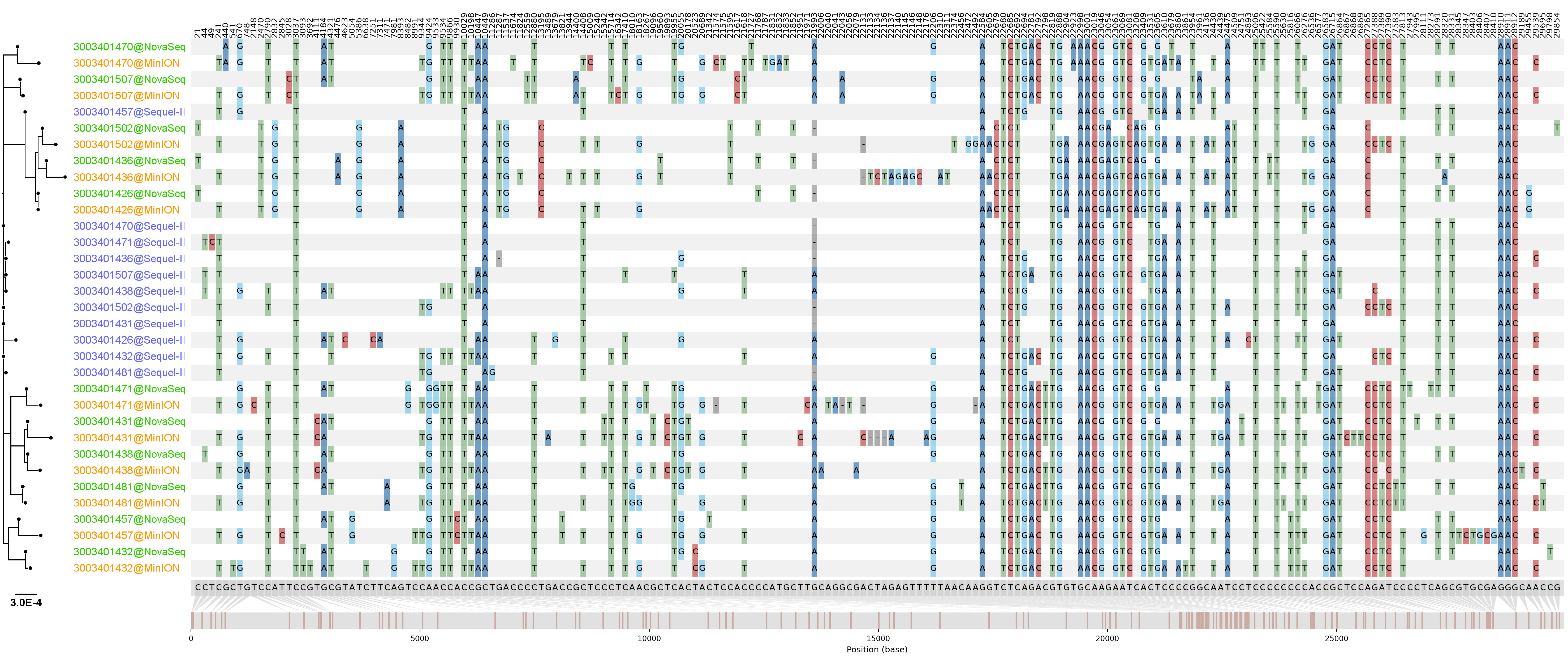

Supplement: Supplementary file 1 [file viruses-17-00584-s001.zip › Figure S3.png]
